# Experiencing modular adaptive decentralized water security systems in southern Pakistan and implications for public health and nutrition: a qualitative exploratory study

**DOI:** 10.3389/fpubh.2026.1780817

**Published:** 2026-05-01

**Authors:** Farooq Ahmed, Najma Iqbal Malik, Razia Anjum, Jam Bilal Ahmad, Sidra Zia, Haleama Al Sabbah, Hasan Dincer, Serhat Yüksel

**Affiliations:** 1 Department of Anthropology, The Islamia University of Bahawalpur, Bahawalpur, Pakistan; 2 Department of Psychology, University of Sargodha, Sargodha, Punjab, Pakistan; 3 Department of Psychology, Academic Centre, Bath Spa University, Ras al Khaimah, United Arab Emirates; 4 Taxila Institute of Asian Civilization, Quaid-i-Azam University, Islamabad, Pakistan; 5 Department of Public Health, College of Health Sciences, Abu Dhabi University, Dubai, United Arab Emirates; 6 School of Business, Istanbul Medipol University, Istanbul, Türkiye

**Keywords:** MAD water infrastructure, Pakistan, public health nutrition, qualitative, social justice

## Abstract

**Introduction:**

This study attempted to explore the perception and experiences of facilitators and challenges in the implementation of modular, adaptive, and decentralized water infrastructure in the community. It also aimed to explore the association between social justice in the implementation of MAD water infrastructure and its potential in improving health and nutrition outcomes in vulnerable communities.

**Methods:**

This exploratory study was conducted in two water-insecure regions in South Punjab, Pakistan. These regions include: (1) Cholistan Desert in Bahawalpur Division, and (2) Western foothill region in Rajanpur District in D.G. Khan Division. The qualitative data were gathered through semi-structured interviews with 20 key individuals, group discussions involving 27 participants, and direct observations. These methods were used to gather data from key stakeholders in water management, communities, and experts in public health.

**Results:**

Overall, five themes and 10 sub-themes were identified. The results showed that inequality in water distribution and accessibility among different socioeconomic groups and genders, as well as private water vendors, hinder water accessibility. Water governance is also marred by elite capture and politics, which has led to a lack of equity in water distribution. Large-scale water infrastructure has also led to a lack of trust in how water is managed. While decentralized, local solutions like small dams, rainwater harvesting, solar technologies, wastewater reuse, and ponds were seen as good options, their success depended on maintaining local capacity and ownership. Where better solutions, such as rainwater harvesting, exist, there are no waterborne diseases and healthy children. While the benefits of the MAD water solutions are high, there are structural barriers to affordability and technical expertise. Many of these solutions prove to be short-lived without external financial and training support.

**Conclusion:**

The study argues that new solutions for the MAD water system need to be ethical, approachable, participatory, gender-sensitive, and politically unbiased.

## 1 Introduction

Household water insecurity, i.e., the absence of sufficient, consistent, inexpensive, and harmless water, which, in addition to harming physical wellbeing, also influences mental wellbeing as well as other social activities (1), is not only an environmental issue, as commonly perceived, but also a structural and administrative issue in the context of water governance, which differs in different regions of the world. In the Global North, as well as in the United States, a centralized water system is predominant, though there exist gaps in the service in the rural as well as desert regions, where service is underserved with small, decentralized, less resilient, and less regulated systems in place (2, 3). In the Global South, countries such as Jordan, Egypt, India, as well as Sub-Saharan countries, have also implemented decentralized or participatory models, though they also seem to be facing certain issues, as noted in reference (4–6).

The macro-level water situation in the country impacts the micro-level water distribution for the southern marginalized regions. The existing water system (based on barrages and permanent headworks across the rivers) in Pakistan is deep-rooted in colonial times. The British developed canal colonies, replacing the irrigation system of seasonal or inundation canals, and ended the traditional harvesting system (7). In Pakistan, after independence in 1947, the landholdings of influential people played an important role in the management of water resources (8). Poor and marginalized low-income groups frequently remain unable to access an adequate quantity of water (9). For example, in Southern Punjab districts, the canal water is provided by the bureaucracy only for 6 months, whereas in other regions of Punjab province, it flows throughout the year (10). Tenant farmers at the tail end of canals often complain of being disadvantaged in terms of water access. Underground water in many areas is brackish, and therefore, the problem of household water access emerges as a significant concern. In South Punjab, marginalized remote desert areas such as Cholistan and hilly areas adjacent to Suleiman Mountains often remain deprived of an already scarce water resource due to such regional disparities (10, 11).

Water crises need political as well as ethical considerations. Water diplomacy is important not only for managing transboundary tensions but also for promoting equitable domestic allocation (12–14). Centralized approaches mostly ignore equity and social inclusion (15). Informal water markets do not obey distribution justice values. Privatization and elite capture of water resources further intensify the stigma, stress, and conflict associated with water insecurity (16–20) and aggravate health and nutrition outcomes owing to higher costs (21), wait times, and poor water quality (22). Water’s moral economy (communities struggle against commodification and seek rights to fair access) is a social justice issue (23, 24).

Water demands can also be addressed by an alternative strategy known as “Modular, Adaptive, and Decentralized” (MAD) systems. The use of MAD Infrastructures is being proposed as a sustainable alternative to traditional water infrastructure. MAD strategies emphasize flexibility, community responsiveness, and resilience to climate change, economic shocks, and infrastructure failure (25–28). MAD systems are capable, but not inherently equitable or effective in all settings. Examples of such include rainwater harvesting, solar-powered pumps, community-managed supply, and small-scale wastewater reuse (29–35).

The notion of environmental justice and the social determinants of health framework are unified, and the various ways through which water insecurity affects health and nutrition outcomes are recognized. Water insecurity is a social issue related to water access, which has a direct influence on health equity (36, 37). We imply that MAD systems are not neutral technology; rather, their ability to influence health outcomes depends on their interaction with existing power structures. Such a theoretical underpinning will guide our subsequent analysis on elite capture and community engagement (38, 39). Water insecurity has an immediate influence on women’s time, labor, and caregiving. MAD systems can reduce the burden on women and disadvantaged groups if implemented fairly. This might lead to improved nutrition and health outcomes, as well as developing its ability to adapt to climate change (40, 41). Along with resolving technological and operational concerns, as well as governing technological and political-economic limitations, building trust is an equally important issue.

Despite advances in the knowledge and technical application of MAD water infrastructure, research on power and inequity, and their consequences for marginalized people, remains limited. Solving the ‘structural barriers to their adoption’ is imperative because health and nutrition are closely connected to social justice in water management. Our research, therefore, aims to (1) explore the enablers and barriers in implementing MAD water systems, and (2) analyze how social justice and equity in MAD water systems are associated with food and nutrition security and improved health outcomes for susceptible communities.

## 2 Materials and methods

### 2.1 Study settings

Two semiarid water-deficient areas of Southern Punjab were considered for the study. First, the Cholistan Desert area of Bahawalpur Division and then the Western Foothills area of Rajanpur District, D.G. Khan Division. The regions were included based on similarity in terms of water insecurity conditions. The areas are highly susceptible to water scarcity, have high poverty rates, and experience adverse health or nutritional consequences, making them suitable for studying MAD systems under severe environmental conditions (42). Cholistan sustains a historic, nomadic, and semi-nomadic pastoralist community of about 0.3 million. It has a hyper-arid climate (<200 mm of precipitation/year) with no permanent rivers, necessitating dependence on ephemeral rainwater ponds (tobas), hand-dug wells, and purchased water. It was once a civilization, nearly 4–5 thousand years old, situated near the River Hakra. With a population of ~ 2 million, Rajanpur is mostly rural and semi-desertic (205 mm rain/year) (43). The Indus River canal system provides only a portion, while groundwater is predominantly brackish and not potable. The Western areas of Rajanpur district are severely water insecure and dependent on, as well as vulnerable to, flash floods from the Suleiman Mountains.

### 2.2 Sampling, inclusion criteria, and data collection

Purposive sampling approach was used to select key stakeholders who were either directly involved in or influenced by water management activities, such as: (1) people from the community, such as mothers and farmers, (2) government officials such as water engineers and managers, (3) individuals from NGOs such as community workers and volunteers, and (4) people from health and nutrition departments and officers/experts. This approach was chosen to ensure that participants with rich information were included to provide detailed responses to the research questions, rather than for statistical representativeness (44). The inclusion criteria for community members were being a primary decision-maker or a household water collector and residing in the community for more than 5 years. Participants were recruited through community gatekeepers, and no eligible individuals declined to participate, except for a few who exhibited evasion during the interviews. We recruited participants through community gatekeepers such as active community members, local councilors, and schoolteachers.

Recruitment involved four groups, each from both the demand and supply sides of key stakeholders, and the final sample was based on sufficiency or practical considerations. In total, 20 semi-structured interviews were conducted: 7 with water officials, 8 with community members (including community workers), and 5 with public health and nutrition experts. Each interview lasted nearly 60–90 min, conducted either in person at respondents’ places or by phone. In addition to 20 semi-structured interviews, 5 focus group discussions were conducted in local villages to understand water insecurity, MAD water use, and their impacts on physical and mental wellbeing. Each FGD lasted 1–2 h, during which 5–6 participants were allowed to participate (Table 1). Of the 5 FGDs, 2 were organized with mothers of female gender, and 3 with male farmers in the village. They clarified the rationale for group size (6–7), time (1–2 h), and the total number of participants (*n* = 27).

**Table 1:** Details of study respondents.

Description of semi-structured interview and FGDs	Number of respondents (n)
Semi-structured interviews with officials	07
Semi-structured interviews with the community notables	08
Semi-structured interviews with health and nutrition experts	05
5 FGD with community members	27
Total participants of interviews & FGDs	47

The total number of unique participants in both qualitative data collection methods was 47. Twenty individuals (*n* = 20) were interviewed using a semi-structured interview guide, and 27 participants took part in five focus groups. Our qualitative exploratory design was not intended for generalization or statistical representativeness; therefore, a small sample size for an exploratory qualitative study was considered appropriate. To obtain diverse perspectives from various stakeholders, we required narratives and experiences. For this purpose, we purposively selected women, farmers, and local leaders for discussions. To ensure participants’ comfort and expression, the lead researcher and trained research assistants used local languages (Saraiki and Urdu) during interactions. We audio-recorded all verbatim responses and transcribed and translated them into English for our analysis.

### 2.3 Interview guide development

A semi-structured interview guide was developed to explore respondents’ experiences and perspectives concerning water access, water governance, and health and nutrition outcomes of water use. The guide explored local water-related concerns, the role and effectiveness of MAD water infrastructure in addressing these challenges, and the types of MAD infrastructure currently used by communities. We also examined respondents’ experiences with the application and management of these systems, including the barriers they faced and the strategies they used to overcome them.

Moreover, the guide explored the influence of social relationships and political affiliations, as well as institutional support, on the implementation and sustainability of MAD infrastructures, and the extent of governmental support for safeguarding social justice during their implementation. Finally, the semi-structured interviews asked about the perceived impacts of MAD infrastructures on public health and nutrition in marginalized communities, with special attention to outcomes associated with water and food security, water, sanitation, and hygiene (WASH), infant and young child feeding (IYCF), breastfeeding practices, and maternal mental health.

### 2.4 Data analysis

The preparation of the interview guide was informed by a deductive approach. We designed a questionnaire using existing literature. However, the data analysis was accomplished using an inductive approach. Following Braun and Clarke’s framework, we employed a qualitative data analysis procedure in multiple phases. In the first step, we familiarized ourselves with the data by frequently reading it. We carefully cross-checked all translations from the local language into English. It was ensured that the intended meanings and cultural sensitivity were protected. Second, using NVivo software, we made line-by-line coding of the entire dataset. These two authors (FA and NIM) coded the entire qualitative textual data to identify several key concepts. Both authors undertook cross-comparison of the primary codes, and after detailed discussion and mutual understanding, modifications were made to the codes, thereby enhancing the credibility of the coding scheme. Then, both authors discussed how to ensure that themes were firmly grounded in the data. Next, the distinct codes were grouped into broader categories, based on the patterns that appeared frequently. Furthermore, to ensure analytical consistency, we also addressed any inconsistencies in the interpretation. It benefited the improvement of the accuracy of the findings. We collated several codes to arrive at potential themes, which were then reviewed and refined to arrive at the final five themes.

### 2.5 Ethical consideration

The Department of Anthropology at The Islamia University of Bahawalpur, Punjab, Pakistan, provided approval for this study (Number: IUB-ANTH-2024-99-S; Date of Approval: June 5, 2024). In addition, informed consent from all the research participants was obtained only after the study’s nature and purpose were explained. Furthermore, the anonymity and confidentiality were fully secured, and our respondents had all the options to withdraw at any time from the interviews if they felt any issue, evasiveness, or discomfort.

## 3 Results

**Figure 1:**
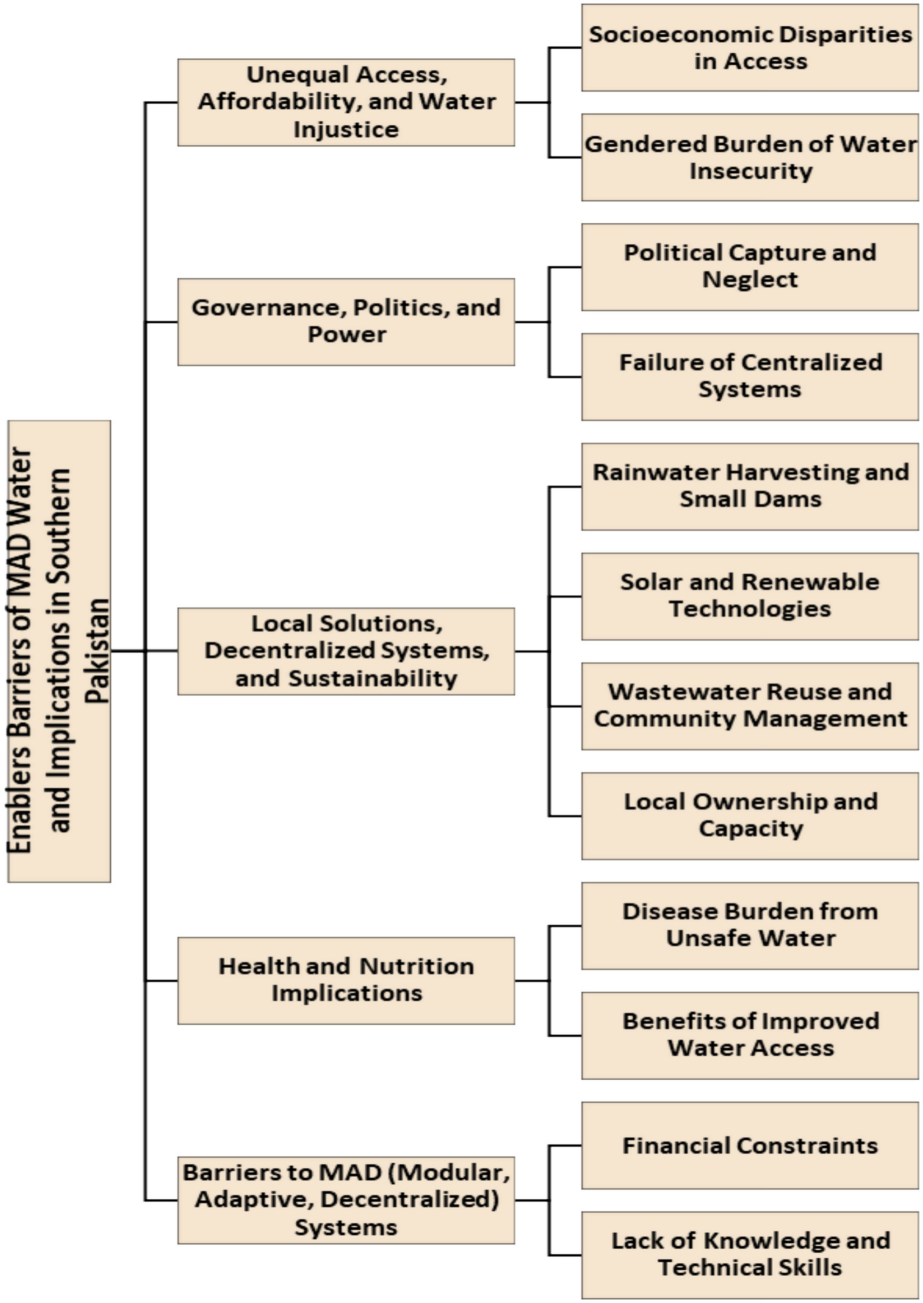
MAD water systems challenges and solutions in southern Punjab, Pakistan.

Using a multi-stage, systematic data analysis method, we identified multiple themes and sub-themes that capture participants’ experiences (Figure 1).

### 3.1 Unequal access, affordability, and water injustice

Deep socioeconomic and gender inequalities determine access to clean water. The underprivileged communities bear the burden disproportionately, and relying on private vendors regularly causes exploitation. The labor of fetching and the cost of water reproduce current inequalities, while women and children bear the brunt of physical and social costs.

#### 3.1.1 Socioeconomic disparities in access

“In search of water and grazing land in Cholistan, the nomadic minority tribes, known as Megwals, have to move with their herds of goats, sheep, and camels. Water is a privilege here, not a basic right, because the poor have to walk far to fetch water. The rich people have the capacity to install and maintain their own tube wells…” (A Minority Nomadic Community Leader, Cholistan)

“Even when water is available from private suppliers, it is beyond our purchasing power. We know it is not always clean, but what option do we have?” (Community Member, South Punjab)

“Clean water has not been available for years… getting it from vendors is the last option, but we cannot afford it.” (Community Member, West Rajanpur)

#### 3.1.2 Gendered burden of water insecurity

“Women of our area often complain that due to water fetching, they become fatigued and cannot properly take care of their children. Also, many young girls frequently remain absent from school because of household labor demands.” (Healthcare provider, Western Rajanpur)

“I have to spend a lot of time fetching water for my children and family. This is what I do every day. The water often affects my children.” (Mother of Three, West Rajanpur)

“Women like me fetch water…use it every day…When it comes to making decisions about water nobody asks us what we think. The men make all the decisions and women are the ones who suffer.” (Mother of Four, West Rajanpur)

“The fact that women are not included in decision making in our village is a problem. This is because of the way things have always been done around here, where men are in charge. When women are not part of the decision making process it makes it harder to make sure that water projects work well and keep working over time.” (NGO worker, South Punjab)

#### 3.1.3 Exploitation and dependence on private vendors

“I admit that the water I vend is not hygienic… This is principally a government duty, not mine.” (Water Vendor, West Rajanpur)

“Big projects cost money but do not reach us. The rich benefit; the poor benefit nothing. Purchasing water is not a solution.” (Community Member, South Punjab)

### 3.2 Governance, politics, and power issues

Regional water governance is characterized by elite capture, political abandonment, and dysfunctional centralized systems. Large-scale schemes bypass marginalized groups, who then have to rely on illegitimate or disorganized arrangements. Restructuring power relations must precede the success of governance reforms. First sub-theme focuses on political capture, patronage, elite influence, and exclusion, while the second addresses the operational and structural limitations of centralized systems, including inefficiency, poor infrastructure reach, and exclusion of peripheral communities.

#### 3.2.1 Political capture patronage, elite influence, and exclusion

“Water governance is a political game and marginalized people… will hardly benefit from whether it is centralized or decentralized until power dynamics are changed.” (Public Health Expert, South Punjab)

“Water-related projects often become lobbied. If there is no benefit to powerful elites, the likelihood of getting funding is lowest.” (Local Politician, South Punjab)

#### 3.2.2 Operational and structural limitations of centralized systems

“The centralized system needs a lot of investment, but political will is lacking. It is outdated and wasteful.” (Water Management Official, South Punjab)

“Big projects cost money but do not reach us. The rich benefit; the poor benefit nothing.” (Community Member, South Punjab)

Water governance, as captured by the powerful elite, excludes marginalized groups. Centralized or big projects benefit the rich, as big dams or massive pipeline schemes often stop near privileged areas and ignore marginalized communities because the budget is deferred due to the high cost of widening the final connections to peripheral or poor communities. Large landlords in South Punjab, or communities in central Punjab at the head of the system, receive adequate and reliable water; nevertheless, pastoralists and poor villages at the “tail-end farmers” receive a minimal fraction. Large-scale schemes are not suitable for all and often raise suspicion regarding state-driven water solutions.

### 3.3 Local solutions, decentralized systems, and sustainability

Respondents remarked that solar pumps, small dams, and rainwater harvesting do not require huge financial investment and are easy to install in various contexts; however, to make these systems sustainable, communities need governmental support, technical training, and scaling-up policies.

#### 3.3.1 Rainwater harvesting and small dams

“A small dam at our village has more chances of being a successful project… Food production and a secure water situation would definitely protect against malnutrition.” (Community Leader, West Rajanpur)

“Rain comes but it flows away in the desert. If we had tanks in our homes, we would not go far for drinking water.” (Community Member, Cholistan)

“Each flood damages our homes. If small dams were constructed, water could be conserved for crops rather than carrying away our lives.” (Community Member, South Punjab)

“Major canal projects are wasted on us. They take years, cost billions, yet poor people still don’t have water.”

#### 3.3.2 Solar and renewable technologies

“Once… a small solar-powered water pump was installed with government help… women no longer needed to walk lengthy distances; however, such projects need scaling up.” (Community Health Volunteer, Southern Punjab)

“Diesel is very expensive. The machine is just idle. If we have solar, we can have water all year round without seeking assistance.” (Community Member, Cholistan)

#### 3.3.3 Wastewater reuse and community management

The reuse of recycled wastewater in agriculture has the potential to improve food and nutrition security by avoiding the exploitation of canal and groundwater resources.

“Why wastewater? If it is treated, it can be used to irrigate crops. We are ready to use it if it is safe.” (Community Member, Rajanpur, South Punjab)

“The people in our village… implemented a small-scale water treatment plant to treat wastewater. We are using the treated water to irrigate our crops, and they look much better.” (Community Leader, Rajanpur district)

#### 3.3.4 Local ownership and capacity

“Locals are managing small water systems… rainwater harvesting systems are working, but these systems do not get promoted on a larger scale without government assistance.” (A Community Activist, Cholistan)

“We’ve tried… to implement a decentralized water management system, but the main issues are technical know-how; hence, some training programs are essential.” (An NGO Worker, South Punjab)

#### 3.3.5 What does not work (centralization and politicization)

In addition to solutions, the participants also highlighted the practices that failed them repeatedly.

“Mega projects regarding water management, like the Kachi canal project, consumed an immense amount of money but did not become successful.” (Water official from Western Rajanpur)

“Though people in Western areas are purchasing water from private vendors, it is still not a panacea because it not only wastes our time and money but also makes us dependent.” (Resident, Western Rajanpur)

Communities skeptical of centralized schemes emphasized that solutions must be ethical and locally controlled. Top-down, large-scale designs hardly reach marginalized groups and even cause more inequities by burdening poor households and women who are already vulnerable. Solutions that prioritize local political patronage over community needs are unsustainable and further fuel mistrust.

### 3.4 Health and nutrition implications

Unsafe water is a direct cause of diarrhea, childhood malnutrition, and maternal health risk, and water scarcity also erodes livestock and food security. Where improved water systems, such as rainwater harvesting, are in place, communities experience fewer waterborne diseases and better child health and nutrition. Having access to good water, therefore, has rippling benefits for family health and nutrition.

#### 3.4.1 Disease burden from unsafe water

“There is a strong nexus of water with people’s health and nutrition… diarrhea and malnutrition are more common in communities with dirty water. Appropriate access to water is a must for handwashing and cleaning, and to prevent diarrhea.” (Public Health Worker, Southern Punjab)

“My child often gets sick because the quality of the drinking water is poor. It’s disturbing his growth.” (Mother of Malnourished Child, Cholistan)

“Our animals are dehydrated and produce no milk when there is a water shortage… All this situation makes things worse not only for animals but also for humans.” (Pregnant Woman, Cholistan)

#### 3.4.2 Nutritional benefits of improved water access

“Here, waterborne diseases were common; then people thought they must implement a rainwater harvesting system… infections reduced significantly.” (Public Health Expert, South Punjab)

“Improved water access decreased the time and energy we spent on childcare. When the care burden is lower, we can engage in more valuable household activities that support family health. Having water near home, we can take care of our families and children, and they become stronger.” (Female Farmer, Southern Punjab)

“There were constant troubles in our lives due to water scarcity…water scarcity reduced exclusive breastfeeding, which ultimately affected infants. This small change brought a big difference in our lives. Being a mother of two children, I can now breastfeed frequently.” (Mother of Two, Cholistan)

“Small solar-powered water pump, increased irrigation, and food production. Nutritional status and food security improved among households that initiated home gardening and prepared nutritious, safe complementary foods for infants. Also, when women get clean water in proximity, they no longer need to walk lengthy distances, which helps them to breastfeed frequently.” (Community Health Volunteer, Southern Punjab)

### 3.5 Barriers to MAD (modular, adaptive, decentralized) systems

Technical and financial challenges hinder these water systems. Upfront expenditures and insufficient training hinder implementation and long-term viability. Investments and capacity development can provide explanations at the local level.

#### 3.5.1 Financial constraints

“The foremost barrier is initial cost, not the availability of technology… such MAD systems will not be in common people’s reach without financial support.” (Water Engineer, Southern Punjab)

“MAD water installation, maintenance, and repair were some serious hurdles for low-income households.” (Community worker, Cholistan)

#### 3.5.2 Lack of knowledge and technical skills

“We often hear about rainwater harvesting… but the way they could be used is not very clear.” (Community Member, West Rajanpur)

“Even if technologies are made available, people are not aware of how to keep them running. Without technical know-how… projects do not last long.” (Local Development Worker, South Punjab)

The claims of decentralized water systems are high, but affordability and insufficient technical expertise remain real challenges. Most of these endeavors can become viable once these barriers are overcome.

## 4 Discussion

Our findings revealed that access to water is shaped by power relations, in which marginalized groups often fail to become beneficiaries. Low-income communities in the periphery rely on unsafe and expensive private vendors. Evidence indicated that in Southern Punjab, canals’ water is available for less than a year; however, water in other regions of Punjab is accessible throughout the year (43). This fact exemplifies how eco-governmentality and political ecology were socially and culturally established through historical inequalities. These disparities exacerbate water insecurity and continue to worsen health and nutrition outcomes, particularly among vulnerable populations. To this end, water insecurity cannot be removed from further intrinsic structural violence, fueling poverty and ill health.

Respondents continued to explain how the low-income marginalized communities are forced to rely on informal water markets where they purchase expensive and dirty water from street vendors. This aligns with studies elsewhere indicating that the informal market, rather than alleviating scarcity, widens inequities and targets poor individuals (20). Such markets capture the paradoxes of neoliberal water regulation: state withdrawal creates space for private actors in public spaces, but, in its absence, without regulation, subjects the public to higher charges for worse service. Wutich et al. argued that the provision of minimum access is insufficient; negative health and nutrition status require water justice to address the structural impediments faced by citizens (16).

Our analysis reveals that water scarcity in Southern Punjab is a social construction, in which rules of canal operations and regimes of allocation produce unequal access (45, 46). This neoliberal water governance (47) necessitates marketized solutions, such as informal vendors and private markets, that tend to shift costs onto the poor. Our respondents’ descriptions of reliance on expensive vendors and elite appropriation thus directly correspond to scholarship on informal water markets and the unequal social effects of water commodification (7).

Our results showed that some communities have successfully managed MAD systems with collective action. However, some villages failed where this condition did not exist, and prevailing power hierarchies were already reinfected. In these cases, the environmental justice framework uncovers interactions with local power relations. Drawing on comparative research on water justice and household water insecurity (48, 49), our study examines distributive and procedural justice and yields distinct results across populations. Our results specify that the technical feasibility of MAD systems is sufficient but not a mandatory condition for equity and justice. Institutional reforms in the sponsorship and marketization of technologies require comparable political-economic configurations.

Community members emphasized that successful systems require individual participation in planning, implementation, and maintenance. This is attested by scholarship on participatory development and community-led governance (50). Women in households are excluded from decision-making and from gender-sensitive water governance (51). Despite technological interventions, their exclusion from decision-making bodies and processes undermines the success and sustainability of decentralized water projects (52).

This study shows a convincing association between water security and public health and nutrition implications. Findings showed that the absence of clean water caused waterborne diseases, malnutrition among children, and psychological distress in pregnant and lactating women. Whereas rainwater harvesting and solar pumps infrastructure helped reduce contamination, improve maternal diet, and mitigate stress (40). Appropriate access to water, as emphasized by attendants, is crucial for hygiene, food security, and overall health. Pathways linking improved water access to health operate through multiple mechanisms. First, when water for hygiene (handwashing, cleaning) was available, waterborne diseases such as diarrhea were eliminated through reduced pathogen exposure. Second, nutritional status improved among households that practiced home gardening and prepared nutritious, safe complementary foods for infants. Third, improved access to water reduced the time and energy women spent on childcare. When the care burden is reduced, women can engage in additional productive household activities that support family health (30, 53, 54).

Finally, the interviewees mentioned scalability challenges for MAD systems, including a lack of technical expertise, cost, bureaucratic administration, and hostility from government authorities. The initial investment cost is strongly discouraging for low-income households, echoing the broader observation in the literature that decentralized frameworks are ineffective when the financial burden is placed on marginalized communities (55, 56). This is once more reflective of the dangers of neoliberal decentralization—states’ withdrawal most adversely affects marginalized communities. Thus, the emancipatory potential of MAD approaches depends on sustained institutional investment, political will, and redistributive policies to prevent marginalized voices from being swamped. Sustainable water for better health outcomes in the context of climate change in Pakistan requires political reforms to implement locally adaptive water management systems and decentralized governance (57–61). It must be made clear that it is not a replacement for state responsibility; rather, a hybrid governance of community-managed MAD systems could be amalgamated within a supportive state framework. The research suggests that a community water fund, along with capacity-building workshops, ought to be initiated to address technical and financial aspects, as well as inclusiveness, participation, and gender issues, to prevent elite capture in water management.

### 4.1 Strengths and limitations

There are certain limitations to this study. The research findings from water-scarce regions of two districts of South Punjab are not representative of the country as a whole. Moreover, it must be admitted that researchers’ reflexivity and positionality might have influenced the coding and interpretation of findings to some degree. The research also has some strengths. For example, it offers a diverse and rich perspective from officials, citizens, and healthcare practitioners. Moreover, it is the first-ever attempt to link alternative modular, adaptive, and decentralized water systems to health and nutrition in the underprivileged regions in Pakistan. This research also adds to the international debate on water justice, arguing that equitable and inclusive decentralization is indispensable for a water-secure future.

## 5 Conclusion

The research aims to investigate the enablers and the challenges faced in implementing MAD water systems in the water-insecure regions of South Punjab, Pakistan. It also examines the role of social justice and equity in the governance of water and its effect on the health and nutritional status of vulnerable populations. According to the results, the implementation of MAD water systems in vulnerable regions of Pakistan’s Southern Punjab was affected by social inequality, social participation, and governance systems. Despite the immense potential of the MAD systems to reduce the workload of women, improve the health and nutritional status of the population, and improve the food security of the people, there are challenges to overcome, including financial, technical, and political. There are, for instance, cases of successful rainwater harvesting system implementation, but to achieve the desired impact, government support and the inclusion of people’s voices must be sought. Improving these elements would make MAD water interventions both sustainable and socially just approaches to water security for marginal communities.

## Data Availability

The original contributions presented in the study are included in the article/supplementary material, further inquiries can be directed to the corresponding authors.
